# Synthesis of the ZnTiO_3_/TiO_2_ Nanocomposite Supported in Ecuadorian Clays for the Adsorption and Photocatalytic Removal of Methylene Blue Dye

**DOI:** 10.3390/nano10091891

**Published:** 2020-09-21

**Authors:** Ximena Jaramillo-Fierro, Silvia González, Hipatia Alvarado Jaramillo, Francesc Medina

**Affiliations:** 1 Departamento d’Enginyería Química, Universitat Rovira i Virgili, Av Països Catalans 26, 43007 Tarragona, Spain; francesc.medina@urv.cat; 2 Departamento de Química y Ciencias Exactas, Universidad Técnica Particular de Loja, San Cayetano Alto, Loja 11-01-608, Ecuador; sgonzalez@utpl.edu.ec (S.G.); hcalvarado@utpl.edu.ec (H.A.J.)

**Keywords:** clay, ZnTiO_3_-TiO_2_, photocatalysis, adsorption, methylene-blue

## Abstract

Currently, the study of semiconductor materials is very promising for the photocatalytic remediation of hazardous organic substances present in the air and water. Various semiconductors have been investigated in this interesting photo-assisted methodology, among them metal oxides such as ZnO, TiO_2_ and their derivatives. In this study, ZnTiO_3_/TiO_2_ was synthesized by the sol-gel method using Ti(OC_3_H_7_)_4_ and Zn(CH_3_COO)_2_ · 2H_2_O as reagents. The role of several conditions such as synthesis temperature and TiO_2_:ZnO proportion on the morphology and purity of compounds obtained was studied, and the suitable conditions for the synthesis of photocatalysts were determined. Various techniques were used to conduct a systematic investigation on the structural, morphological, and photocatalytic properties of ZnTiO_3_/TiO_2_. Scanning Electron Microscopy (SEM) images show that ZnTiO_3_/TiO_2_ have a typical particle size of approximately 100 nm with a quasi-spherical shape. The adsorption and photocatalytic activity were investigated by the decolorization of Methylene Blue (MB) as an organic contaminant under UV irradiation both in TiO_2_ and ZnTiO_3_/TiO_2_ supported over some Ecuadorian clays. The materials evaluated were prepared in the shape of 0.2 cm (diameter) and 1.0 cm (length) cylindrical extrudates. The degradation percentage of MB obtained was 85% approximately after 150 min of irradiation. The results obtained allow us to conclude that these synthesized materials can be used as adsorbents and photocatalysts.

## 1. Introduction

The presence of pollutants in wastewater has a dangerous potential impact on the environment and on health. In fact, water and wastewater treatment is a major problem in our society [1]. Several industries use large amounts of dyes for their end-products. For instance, the textile industry uses more than 10,000 types of dyes. This industry discharges 2% to 20% of its dyes as aqueous effluents [2]. Many of these substances are not biodegradable and pollute the aquatic environment [3], and some of them have a carcinogenic effect [4]. Hence, it is necessary to treat the effluents before discarding them.

There are several technological processes to remove persistent pollutants from water [1,5,6,7,8]. Among these processes, photocatalysis is appreciated for its effectivity [9,10]. Photocatalysis is an Advanced Oxidation Process (AOP) that requires a semiconductor [11] that interacts with photons and the adsorbed composite through surface charges. The interaction produces radical species that decompose the adsorbed composite through redox reactions [12].

Titanium dioxide (TiO_2_) is a widely used semiconductor for photocatalytic applications. TiO_2_ has low toxicity, low cost, is chemically stable, and is an excellent oxidant [13]. TiO_2_ is a photocatalyst with a band gap value of 3–3.2 eV, which depends on its crystalline structure. Furthermore, TiO_2_ is interesting due to its efficiency in wastewater treatment [14].

On the one hand, TiO_2_ is the most popular photocatalyst; nevertheless, it has low sensitivity to the visible light spectrum. On the other hand, zinc oxide (ZnO) is also inexpensive, but has high activity as a photocatalyst in visible light [15]. This oxide has a band gap energy (3.2 eV) similar to that of TiO_2_. Both oxides can improve their own photocatalytic activity through TiO_2_-ZnO coupling [16]. This improvement is due to a reduced recombination of electron holes. Besides, the greater migration of photogenerated carriers also improves this activity [17].

Synthesis using the sol-gel method of coupled ZnO-TiO_2_ [18] can generate some Ti-Zn mixed oxides, such as Zn_2_TiO_4_, Zn_2_Ti_3_O_8,_ and ZnTiO_3_, among the main derived products [19,20]. Some impurity phases can also appear in the final products [21,22]. During the synthesis, the transformation of the crystalline phases presents different products, which depend on the initial molar ratio of the reactants and the calcination temperatures. The presence of anatase and rutile can also affect the behavior of the phases [23].

Ti-Zn mixed oxides are low-cost materials and harmless to the environment [21]. These materials are important due to their technological applications [24]. Ti-Zn mixed oxides can act as pigments, photodetectors, dielectric materials, sensors, and light emitting diodes [25]. Several studies also show their wide use for the photocatalytic decomposition of organic compounds since their band gap has a value of 3.06 eV [26,27,28,29].

Although the use of photocatalysts is an interesting alternative for wastewater treatment, their recovery at the process-end limits their practical use. The structured supports offer an alternative to keep the photocatalyst on its surface [30]. Thus, the separation stage becomes unnecessary. The literature shows various materials that serve as supports [31]. Among them are silica, ash, zeolites, active carbon, and clays [32,33]. Clays and materials derived from clay represent a potential alternative. These materials have high chemical and mechanical stability [34]. Besides, they have a great adsorption capacity due to their high surface area [35]. Clays are low-cost materials and offer an interesting route to revalue local resources [36].

The present study reports the synthesis of the ZnTiO_3_/TiO_2_ composite supported in several Ecuadorian natural clays, as well as its efficiency in the adsorption and photocatalytic degradation of methylene blue (MB) in aqueous effluents. The adsorption and photocatalytic degradation of methylene blue were determined by the decolorization of the dye and were quantified by UV-Visible spectrophotometry. The synthesized composites were characterized using X-Ray Diffractometry (XRD), X-Ray Fluorescence (XRF), Diffuse Reflectance Spectroscopy (DRS), and Scanning Electron Microscopy (SEM) techniques.

## 2. Materials and Methods

### 2.1. Materials

All of the reagents used in this study were of analytical grade and used without additional purification: C_3_H_8_O (Sigma Aldrich, St. Louis, MO, USA, ≥99.5%), Ti(OC_3_H_7_)_4_ (Sigma Aldrich, 98%), CH_3_COOH (Sigma Aldrich, 99.8%), HCl (Fisher Scientific, Waltham, MA, USA, 37%), cetyl-trimethyl ammonium chloride (C_19_H_42_NCl) (Sigma Aldrich, 25%), H_2_O_2_ (Sigma Aldrich, 35%), AgNO_3_ (Sigma Aldrich, >99.8%), HNO_3_ (Sigma Aldrich, 69%), Zn(CH_3_COO)_2_ · 2H_2_O (ACS, St. Louis, MO, USA, ≥98%), and C_16_H_18_ClN_3_S · *n*H_2_O (Sigma Aldrich, ≥95%).

### 2.2. Clay Purification

The raw clays were collected from southern Ecuador. The clay samples were ground and sieved to No. 200 ASTM mesh (0.075 mm size). Carbonates of magnesium and calcium were removed using a hydrochloric acid solution (0.1 N) at the ratio of 10 mL/g. The organic matter in the clay samples was removed by oxidation using H_2_O_2_ (33%) in the proportion of 10 mL of H_2_O_2_ for each gram of clay. The samples were constantly stirred for 2 h at room temperature. Subsequently, the samples were centrifuged and washed with distilled water to eliminate Cl^−^ ions, which was verified with a test with AgNO_3_. The clay adsorption sites were activated with nitric acid (0.8 N) using a ratio of 10 mL/g. Activation is a process through which a partially dissolved material is obtained, which has greater surface acidity, porosity, and surface area [37]. The activated clay samples were centrifuged, washed with distilled water, and dried at 60 °C for 24 h.

### 2.3. Synthesis of the ZnTiO_3_/TiO_2_-Clay Composite

The ZnTiO_3_/TiO_2_ photocatalysts were synthesized following a modified sol-gel method described for other nanocomposites [38,39]. Several TiO_2_/ZnO molar ratios were tested (9:1, 4:1, 3:1, and 2:1). Different calcination temperatures (500, 600, 700, 800, 900, and 1000 °C) were also tested to obtain the optimal photocatalyst synthesis conditions. Afterwards, the synthesis process was repeated in a clay solution as follows: Clay (1 g) was dissolved in isopropyl alcohol (iPrOH) (10 *w*/*w*%) under stirring for 24 h to achieve homogenous solution. Subsequently, a quantity of titanium (IV) isopropoxide (TiPO) in iPrOH (70 *v*/*v*%) was added to the solution at room temperature, using a ratio of 10.5 mmol of Ti per gram of clay. Previously, a solution formed by Zn(acet), water, and iPrOH was slowly added, using the optimal ZnO/TiO_2_ molar ratio. The amount of water had a 50 *v*/*v*% iPrOH/water ratio and was determined by stoichiometry, being the amount necessary to hydrolyze the TiPO molecules. The synthesis was performed at room temperature. The reaction system was additionally stirred for 30 min. The mixture was kept under stirring at room temperature for another 30 min after the formation of a precipitate. The precipitate was dried at 60 °C for 24 h and calcined at the optimum temperature for 4 h. Finally, the products were cooled at room temperature. To obtain clay supported TiO_2_, the procedure described above was repeated for a final ratio of 10.5 mmol Ti per gram of clay but without the addition of ZnO.

### 2.4. Preparation of the Supported Photocatalysts

For the evaluation of the solid materials, cylindrical extrudates with approximate dimensions of 0.2 cm in diameter and 1.0 cm in length were prepared. The preparation of these solids was carried out by mixing the solid materials with water (approximately 35%) to form a mixture with good plasticity. This mixture was extruded with a 2.5 mm diameter syringe. The extrudates were dried at 90 °C for 2 h and finally calcined at 500 °C for 8 h.

### 2.5. Characterization

The synthetized materials were characterized using a JEOL JSM 6400 scanning electron microscope (SEM) (JEOL, Peabody, MA, USA). X-Ray Fluorescence (XRF) measurements were recorded in a Bruker S1 Turbo SDR portable spectrometer (Bruker Handheld LLC, Kennewick, WA, USA), using the Mining Light Elements measurement method. The X-ray diffraction (XRD) measurements were recorded in a Bruker-AXS D8-Discover diffractometer (Bruker AXS, Karlsruhe, Germany) equipped with a vertical *θ-θ* goniometer, a parallel incident beam (Göbel mirror), and a HI-STAR General Area Diffraction Detection System (GADDS) (Bruker AXS, Karlsruhe, Germany). The X-ray diffractometer was operated at 40 kV and 40 mA to produce the Cu Kα radiation (1.5406 Å). Data were recorded from 5–70° in the 2*θ* range. The identification of the crystal phases was obtained by comparison of the XRD profile with the ICDD (International Centre for Diffraction Data, release 2018) database. The determination of the specific surface area of the solids (m^2^/g) was carried out in the ChemiSorb 2720 equipment (Micromeritics, Norcross, GA, USA), by nitrogen adsorption at the temperature of liquid nitrogen (−196 °C) with a 30% gas mixture of N_2_ diluted in He. UV-Vis diffuse reflectance spectrum (DRS) of the photocatalysts were obtained on an UV-Vis spectrophotometer Thermo model: Nicolet Evolution 201/220 (ThermoFisher, Waltham, MA, USA), equipped with an integration sphere unit using BaSO_4_ as reference. The Chemisoft TPx System (version 1.03; Data analysis software; Micromeritics, Norcross, GA, USA, 2011) allowed to calculate the specific surface area using the BET equation and the single point method.

### 2.6. Adsorption and Photocatalytic Degradation

Heterogeneous photocatalysis experiments were firstly performed at free pH = 8.0 during 150 min, varying the methylene blue solution concentrations (50, 25, 10, and 5 mg/L) and catalyst concentrations (100, 250, and 500 mg/L). In addition, the effect of the ZnO:TiO_2_ molar ratio was examined to select the best catalyst. All the experiments were started with a 30 min adsorption step under dark conditions to obtain the adsorption-desorption equilibrium.

The photocatalytic activity of the compounds was evaluated by the photocatalytic degradation of methylene blue under solar light radiation. Solar light was simulated by a solar box equipped with an air-cooled 1500-W Xenon lamp (Atlas Material Testing Technology, Mount Prospect, IL, USA), which allows 300–800 nm wavelengths to pass through (ATLAS, SUNTEST CPS+). Irradiance was set to 250 W/m^2^. The photocatalytic activity of the compounds was also evaluated by the decolorization of methylene blue under UV-C (254 nm) light radiation to improve the photocatalytic degradation of MB [40].

Typically, 25 mg of catalyst were magnetically stirred in a methylene blue aqueous solution (MB) (100 mL of water containing 5 mg/L methylene blue). The solution was maintained in dark conditions for 30 min. Then, the suspensions were irradiated using solar or UV-C light (100 W germicidal UV-C). The temperature of the photoreactor (25 °C) was controlled throughout the reaction using a cooling circulator air system. The tests were carried out without adjusting the pH = 8.0. The remaining concentrations of methylene blue were determined at 623 nm using a Jenway 7350 spectrophotometer (Cole-Parmer, Staffordshire, UK). The MB removal rate was calculated by absorbance according to the Beer–Lambert law. Samples were drawn at 5 min intervals with a syringe and filtered through a 0.45 µm membrane filter to remove any solid particles interfering with the measurement. All tests were carried out in triplicate using a methylene blue solution blank irradiated with solar or UV-C light to eliminate any photolysis effect due to the light. The decolorization rate was better when using UV-C light for the selected operating conditions; in this way, the tests with the photocatalysts supported on clays were carried out under UV-C light.

The adsorption capacity of the synthetized materials was evaluated by the removal of methylene without light irradiation, using the same protocol that was used during the photocatalytic test.

### 2.7. Reuse of the Supported Photocatalysts

A recycle experiment on photocatalytic degradation of MB by ZnTiO_3_/TiO_2_-Clay, and TiO_2_/Clay was designed to determine the recycling property of these composites. After completing a treatment cycle, the catalysts extrudates were left in quiescent conditions for 60 min to achieve their precipitate. Then, the clear solution was removed from the reaction system and 100 mL fresh MB solution (5 mg/L) was injected into the reaction system, starting the next cycle. The recycle experiment was carried out for five cycles. Each cycle lasted 150 min under UV-C irradiation.

## 3. Results

### 3.1. Characterization of the Samples

#### 3.1.1. XRD Analysis

**Figure 1:**
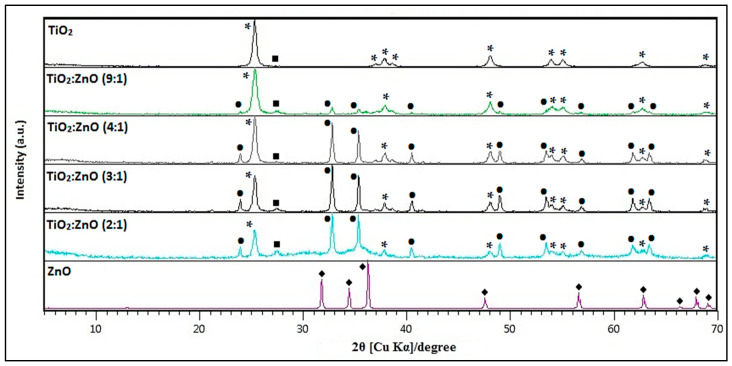
X-Ray Diffractometry (XRD) of ZnTiO_3_/TiO_2_ synthesized at 500 °C with different TiO_2_/ZnO molar ratios. *: TiO_2−a_ (anatase), ■: TiO_2−r_ (rutile), ●: ZnTiO_3_ and ◆: ZnO.

Figure 1 shows Ti-Zn mixed oxides synthesized at 500 °C with different TiO_2_/ZnO molar ratios. The diffraction peaks of ZnTiO_3_ at 2*θ* values of 23.92°, 32.79°, 35.31°, 40.45°, 48.93°, 53.44°, 56.82°, 61.79°, and 63.39° which are assigned to planes (0 1 2), (1 0 4), (1 1 0), (1 1 3), (0 2 4), (1 1 6), (0 1 8), (2 1 4), and (3 0 0), respectively. Similarly, the diffraction peaks of anatase (TiO_2-a_) at 2*θ* values of 25.28°, 36.95°, 37.80°, 38.58°, 48.05°, 53.89°, 55.06°, 62.12°, 62.69°, and 68.76° are assigned to planes (1 0 1), (1 0 3), (0 0 4), (1 1 2), (2 0 0), (1 0 5), (2 1 1), (2 1 3), (2 0 4), and (1 1 6), respectively. Finally, the diffraction peaks of rutile (TiO_2−r_) at 2*θ* values of 27.45°, 36.09°, 41.23°, 54.32°, 56.64°, and 69.01° are assigned to planes (1 1 0), (1 0 1), (1 1 1), (2 1 1), (2 2 0), and (3 0 1), respectively. The ZnTiO_3_-TiO_2_ heterostructure nanomaterial obtained was indexed to an hexagonal phase with unit cell parameters *a* = *b* = 5.08 Å and *c* = 13.93 Å, and space group R-3(148) according to the standard JCPDS card No. 00-015-0591 for the ZnTiO_3_ phase. The TiO_2−a_ species was indexed to a tetragonal phase with unit cell parameters *a* = *b* = 3.79 Å and *c* = 9.51 Å, and space group |41/amd(141) according to the standard JCPDS card No. 01-073-1764, and the TiO_2−r_ phase was assigned to a tetragonal phase with unit cell parameters *a* = *b* = 5.08 Å and *c* = 13.93 Å, and space group P42/mnm(136) according to the standard JCPDS card No. 03-065-0192.

**Figure 2:**
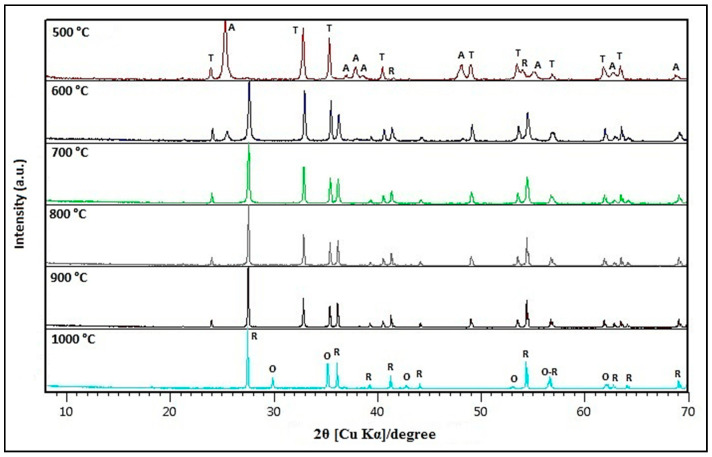
XRD of Zn_x_TiO_y_-TiO_2−a_ synthesized to different temperatures. **T**: ZnTiO_3_; **A**: TiO_2−a_; **R**: TiO_2−r_; **O**: Zn_2_TiO_4_.

Figure 2 demonstrates the effect of the calcination temperature on the formation of different crystallographic phases for the sample prepared with the molar ratio of TiO_2_/ZnO (3:1).

The crystalline sizes of powder samples prepared at 500 °C with the molar ratio of TiO_2_/ZnO (3:1) were calculated based on the main peak using the well-known Scherrer equation [41]:

(1) $$A\;=\;\frac{K\lambda }{\beta \;\cos\theta }$$

where *K* is the shape factor (here, *K* = 0.89), λ is the wavelength of the X-ray beam used (here, *λ* = 0.15406 nm), *θ* is the Bragg angle, and *β* is the full width at half maximum (FWHM) of the X-ray diffraction peak, which was calculated using MDI JADE, version 6; Computer software, Materials Data Inc., Livermore, CA, USA, 2014. The average crystalline size of the phases present in the ZnTiO_3_/TiO_2_ composite were 33.79 (±3.67) and 21.47 (±3.91) nm for ZnTiO_3_ and TiO_2__−a_, respectively.

#### 3.1.2. SEM-EDX Analysis

**Figure 3:**
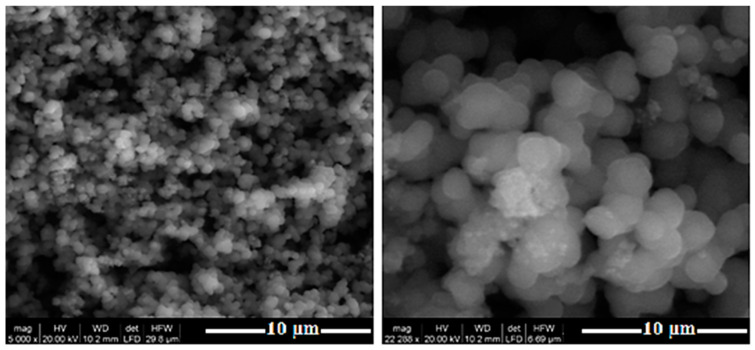
Scanning Electron Microscopy (SEM) images of the ZnTiO_3_-TiO_2−a_ heterostructure material.

Figure 3 shows the SEM image of the ZnTiO_3_/TiO_2_ heterostructure synthesized with a TiO_2_:ZnO molar ratio of 3:1 and calcined at 500 °C. The image shows that the particles have an average particle size of 100 nm, are almost spherical and highly agglomerated.

**Figure 4:**
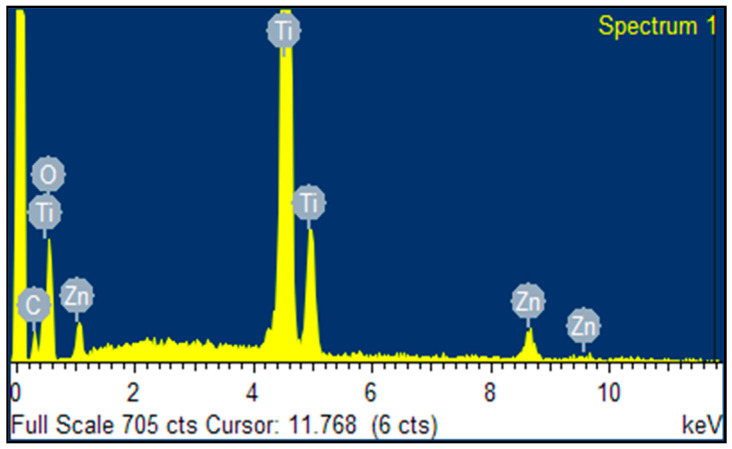
Energy Dispersive X-ray (EDX) spectra of ZnTiO_3_/TiO_2._

Figure 4 shows the Energy Dispersive X-ray (EDX) spectra of the ZnTiO_3_/TiO_2_ heterostructure, indicating the presence of O, Zn and Ti only in the ZnTiO_3_/TiO_2_ heterostructure material. The analyses showed that the heterostructure consisted of C (5.42%), O (33.6%), Ti (54.85%), and Zn (6.13%).

#### 3.1.3. Optical and Photoelectric Properties

**Figure 5:**
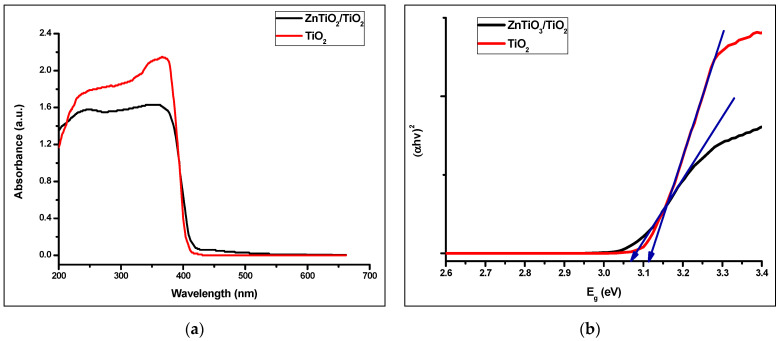
(**a**) UV-vis XRD and (**b**) plots of (*αhv*)^2^ vs. *E*_g_ of ZnTiO_3_/TiO_2_ and TiO_2._

The optical absorption properties of photocatalysts can be characterized using the UV-visible (UV-vis) DRS in the range of 200–700 nm and at room temperature. Figure 5a shows the UV-vis XRD of ZnTiO_3_/TiO_2_ and TiO_2_. Compared to TiO_2_, the visible light absorption intensity of ZnTiO_3_/TiO_2_, at around 400 nm, was improved, suggesting that the ZnTiO_3_/TiO_2_ composite has a better response to visible light. The graphs of (*αhv*)^2^ versus *hv* for calculating the direct band-gap energy (*E*_g_) are shown in Figure 5b. According to Equation (2) [42], the direct *E*_g_ values were 3.07 and 3.12 eV for ZnTiO_3_/TiO_2_ and TiO_2_, respectively. The direct *E*_g_ value calculated for ZnTiO_3_/TiO_2_ can be related to the direct band gap of hexagonal or cubic ZnTiO_3_ compound [43].

(2) $$E_{g}\;=\;\frac{1240}{\lambda }$$

where *E*_g_ is the band-gap energy in the electron volts (eV) and *λ* represents the lower cutoff wavelength in nanometers (nm).

**Figure 6:**
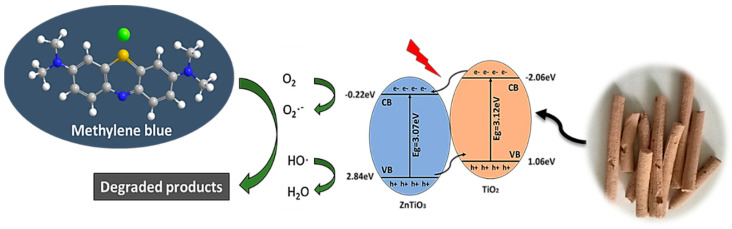
Schematic of electron-hole separation and transportation on the interface ZnTiO_3_/TiO_2_ heterojunction.

In order to analyze the photocatalytic mechanism of ZnTiO_3_/TiO_2_ heterojunction, the potentials of the Valencia Band (VB) and Conduction Band (CB) of TiO_2_ and ZnTiO_3_ were determined, which are important to estimate the flow diagram of pairs of photoexcited charge carriers in heterojunction. The Mulliken electronegativity theory was used to calculate the VB and CB potentials of TiO_2_ and ZnTiO_3_ [41]:

(3) $$E_{\mathrm{CB}}\;=\;\chi -E_{c}-0.5E_{g}$$

(4) $$E_{\mathrm{VB}}\;=\;E_{\mathrm{CB}}+E_{g}$$

where *E*_CB_ and *E*_VB_ are the CB edge potential and VB edge potential, respectively, *E*_g_ is the band gap energy of the semiconductor, *E*_c_ is the energy of free electrons on the hydrogen scale (approximately 4.5 eV), and *χ* is the electronegativity of the semiconductor. The values of ZnTiO_3_ and TiO_2_ were 4.0 and 5.8 respectively. According to the formula above, *E*_CB_ and *E*_VB_ for ZnTiO_3_ and TiO_2_ were (−0.22, +2.84) eV and (−2.06, +1.06) eV, respectively. Figure 6 describes the photocatalytic mechanism of the ZnTiO_3_/TiO_2_ heterojunction.

It was clear that the CB edge of TiO_2_ was more negative than the CB edge of ZnTiO_3_; on the other hand, the VB edge of ZnTiO_3_ was more positive than the VB edge of TiO_2_. Consequently, the electrons excited from the CB of TiO_2_ jumped to the CB of ZnTiO_3_, and the holes generated from the VB of ZnTiO_3_ transferred to the VB of TiO_2_, which caused an efficient separation of *e*^−^ and *h*^+^, and the photocatalytic activity improved due to the ZnTiO_3_/TiO_2_ heterojunction [27,44,45].

#### 3.1.4. XRF Analysis

Clays are minerals that are widely distributed in nature, being produced by the erosion of rocks. The texture and chemical composition of the clays are greatly varied, depending on the presence of organic and inorganic impurities as well as the geological origin of the clays. Table 1 shows the composition of the clays used in the present work; all the clays contained TiO_2_ and Fe_2_O_3_, but Clay_12_ had the highest content of both oxides. Table 1 also shows the presence of other oxides, such as MgO, P_2_O_4_, K_2_O, CaO, MnO, Co_3_O_4_, SnO_2,_ and CeO_2_.

### 3.2. Adsorption and Photocatalytic Degradation

**Figure 7:**
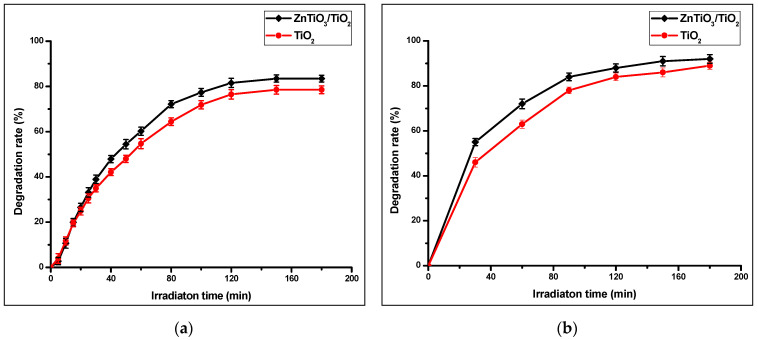
(**a**) Photocatalytic degradation activity of Methylene Blue (MB) for ZnTiO_3_/TiO_2_ and TiO_2_ under irradiation of solar light and (**b**) under UV-C light.

Photocatalysts can degrade organics compounds due to strong oxidizing capacity they exhibit, especially when they are irradiated by light. In this work, the photocatalytic activity of the TiO_2_ and ZnTiO_3_/TiO_2_ photocatalyst was first tested by the degradation of methylene blue (MB) in water, using irradiation with both a) solar light and b) UV-C light. Figure 7 shows the results obtained in the test.

**Figure 8:**
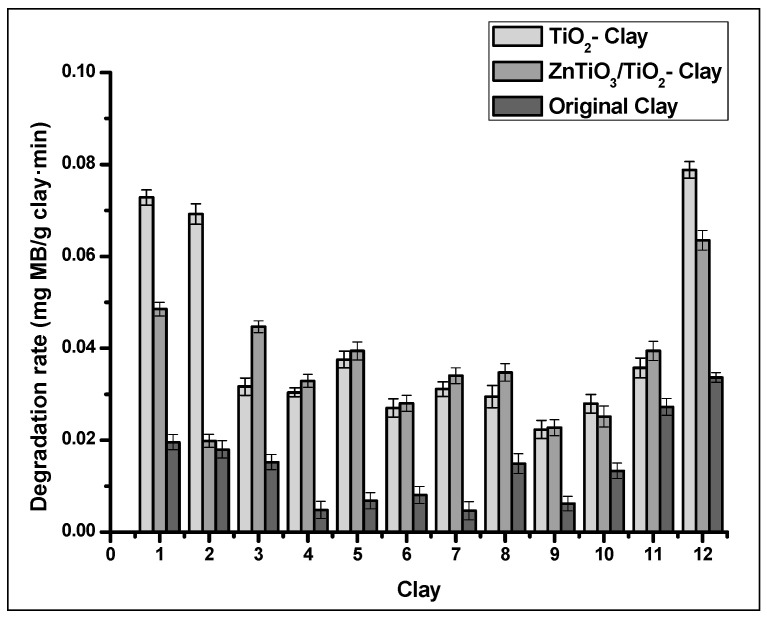
Photocatalytic degradation activity of MB for clay and clay-supported photocatalysts.

Figure 7 shows that the highest removal percentage was obtained with UV-C irradiation; therefore, it was used for photocatalytic degradation tests with photocatalysts supported on clays. Figure 8 shows the photocatalytic activity of the clays and the clay supported photocatalyst. In all tests, the clays improve their catalytic activity with the presence of photocatalysts. ZnTiO_3_/TiO_2_ have higher photocatalytic activity than TiO_2_ in almost all the clays. In this Figure, it is observed that Clay_12_ and the photocatalyst-Clay_12_ composites had the highest photocatalytic activity.

**Figure 9:**
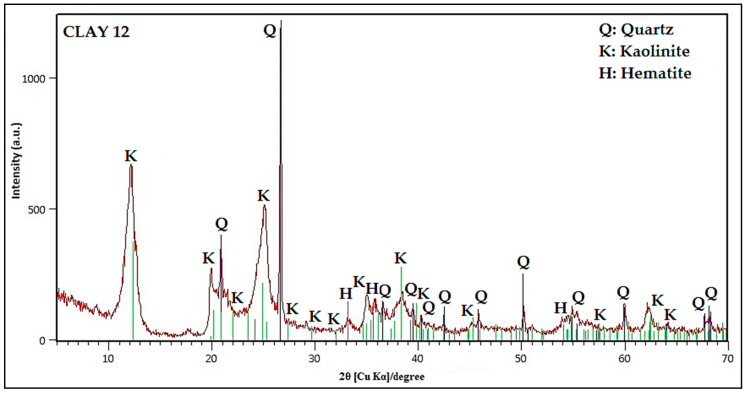
XRD of Clay 12.

Figure 9 presents XRD pattern of Clay_12_, which consists of Kaolinite (K), Quartz (Q), and Hematite (H). This clay has a specific surface area (BET) of 48.8 m^2^/g determined in the Chemisorb 2720 equipment coupled to TPx.

**Figure 10:**
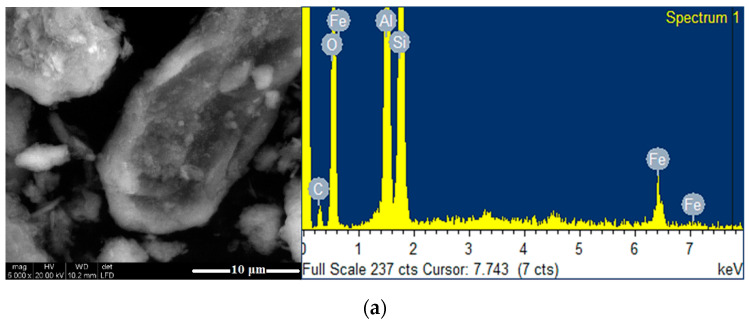

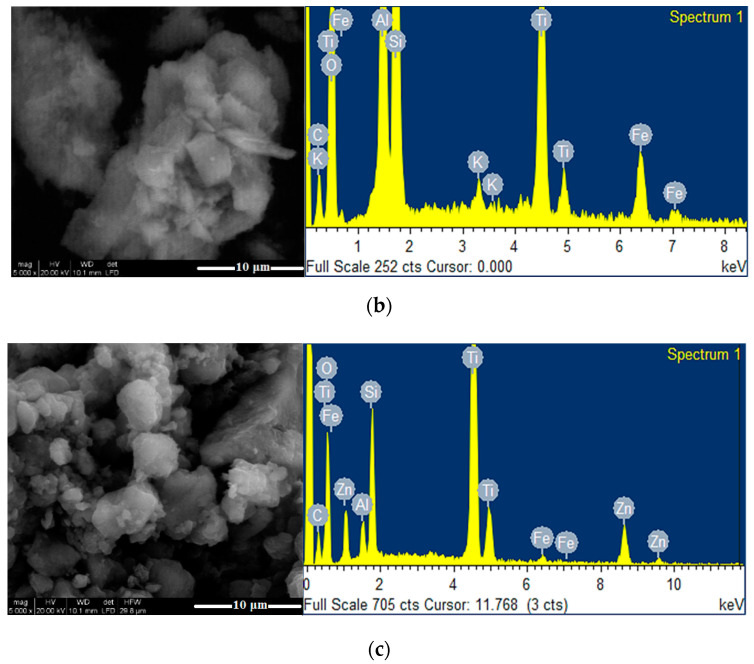
SEM image and EDX spectra of: (**a**) Clay_12_; (**b**) TiO_2_-Clay_12_; (**c**) ZnTiO_3_/TiO_2_-Clay_12_.

The surface morphology of Clay_12_ and of the composites (TiO_2_-Clay_12_ and ZnTiO_3_/TiO_2_-Clay_12_) was investigated by SEM, and the results are shown in Figure 10, from which it can be seen that Clay_12_ has a different surface morphology than composites of photocatalyst-Clay_12_. The surface morphology of Clay_12_ seems as a smooth surface in some parts of the particle, while the composites of photocatalyst-Clay_12_, has some catalysts particles incorporated into its surface (smaller TiO_2_ and ZnTiO_3_/TiO_2_ grains on the outer face of Clay_12_). The EDX spectra confirms that the composites contain an important amount of titanium and zinc, as shown in Figure 10.

**Figure 11:**
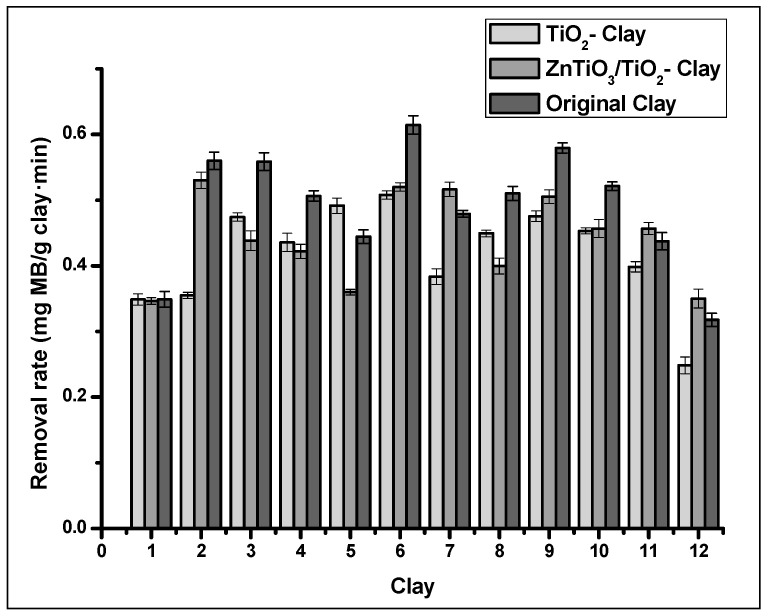
Adsorption capacity of MB for clay and clay-supported photocatalysts.

Figure 11 shows the adsorption results of the clays and clay-supported photocatalysts. Most clays exceed the adsorption capacity of clay-supported photocatalysts. However, TiO_2_ improves the adsorption capacity of Clay_5_ while ZnTiO_3_ slightly improves the adsorption capacity of Clay_7_, Clay_11_, and Clay_12_. In Clay_1_, the presence of photocatalysts did not affect the adsorption capacity of the clay.

**Figure 12:**
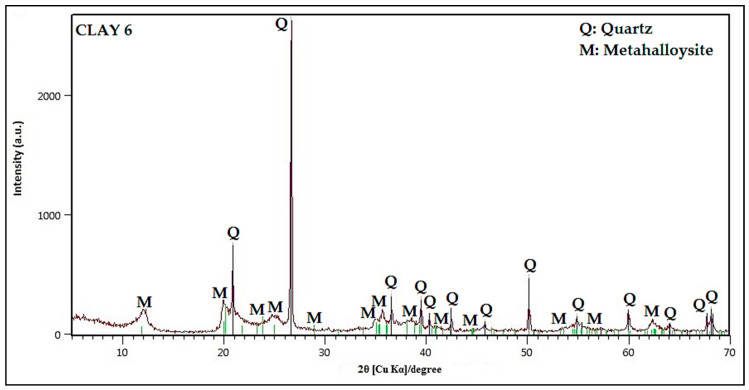
XRD of Clay 6.

In Figure 11, Clay_6_ shows the highest MB removal capacity. The XRD pattern in Figure 12 shows that Clay_6_ consists mainly of Metahalloysite (M) and Quartz (Q), which could also contribute to the high adsorption capacity of the clay. This clay has a specific surface area of 42.8 m^2^/g, which was calculated from N_2_ gas adsorption using the Brunauer–Emmet–Teller (BET) isotherm in the Chemisorb 2720 equipment coupled to TPx.

**Figure 13:**
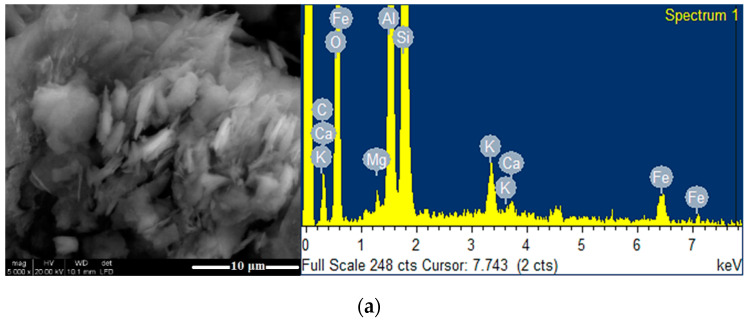

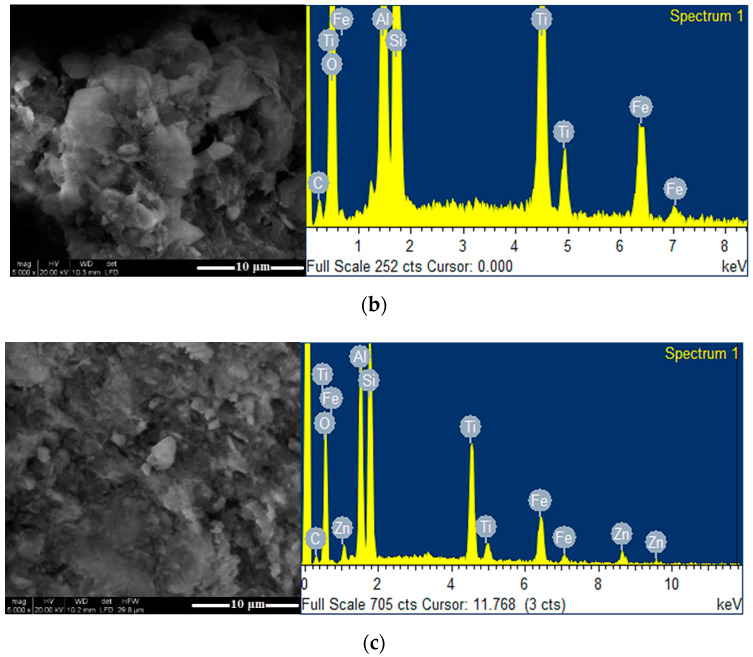
SEM images and EDX spectra of: (**a**) Clay_6_; (**b**) TiO_2_-Clay_6_; (**c**) ZnTiO_3_/TiO_2_-Clay_6_.

Figure 13 show the surface morphology of Clay_6_ and of the composites (TiO_2_-Clay_6_ and ZnTiO_3_/TiO_2_-Clay_6_) examined by SEM. From this Figure, it can be seen that Clay_6_ has a different surface morphology than composites of photocatalyst-Clay_6_. The surface morphology of Clay_6_ seems to be a flake-like structure, while in the composites of photocatalyst-Clay_6_, the surface appears with less flake but with some catalyst particles incorporated (smaller TiO_2_ and ZnTiO_3_/TiO_2_ grains) on the outer face of Clay_6_. The changes in the morphology of Clay_6_ is due to the thermal treatment to incorporate the photocatalysts. This treatment decreased the specific surface area of Clay_6_ to 27.2 m^2^/g and 25.8 m^2^/g when impregnated with TiO_2_ and ZnTiO_3_, respectively. The EDX spectra confirms that the composites contain an important amount of titanium and zinc, as shown in Figure 13.

### 3.3. Reuse of the Supported Photocatalysts

**Figure 14:**
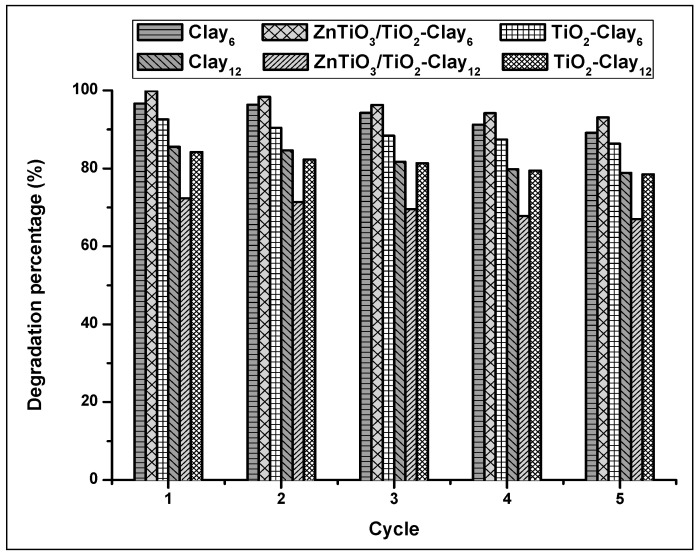
Photodegradation percentage of MB for five successive photocatalytic cycles.

The recyclability and stability of the photocatalysts are important factors for their application in large scale; therefore, five consecutive photocatalytic experiments were carried out for the extrudates of Clay_6_, photocatalyst-Clay_6_, Clay_12_, and photocatalyst-Clay_12_. Figure 14 shows the degradation efficiency of these materials for five cycles.

Figure 14 clearly shows that the percentage of MB removal decreases slightly with increasing cycle times. However, after five cycles, the synthesized materials still have high activity and can efficiently degrade MB in aqueous solution.

## 4. Discussion

### 4.1. Synthesis and Characterization of the TiO_2_/ZnTiO_3_-Clay Composite

In this work, ZnTiO_3_/TiO_2_ nanoparticles were successfully synthesized using the sol-gel method and supported on twelve raw clays of Ecuadorian origin. In the synthesis process when the TiO_2_/ZnO molar ratio was decreased, the formation of TiO_2_ also decreased, but the formation of ZnTiO_3_ increased due to the greater availability to form the heterostructure. The obtained nanomaterial of ZnTiO_3_/TiO_2_ heterostructure had high purity and no other crystalline phases were observed for the sample. This results are consistent with those reported by Bhagwat et al., who synthesized ZnTiO_3_@TiO_2_ at 700 °C using TiO_2_ (P-25) with zinc nitrate as a precursor [28].

Several calcination temperatures were tested for the synthesis of the ZnTiO_3_/TiO_2_ heterostructure. When the heat treatment temperature was increased to 500 °C, TiO_2−a_ (anatase phase) was the primary crystalline phase with TiO_2-r_ (rutile phase) traces. In addition, a hexagonal phase of ZnTiO_3_ was also present in the sample prepared at 500 °C. With a temperature increment from 600 °C to 900 °C, TiO_2−r_ appeared, and TiO_2−a_ disappeared completely. However, the hexagonal phase of ZnTiO_3_ still existed in the sample at 900 °C. This indicates that TiO_2−r_ was transformed from TiO_2−a_, but not by decomposition of ZnTiO_3_. This is in agreement with previous studies which revealed that the phase transition from TiO_2−a_ to TiO_2−r_ occur in a temperature range of 600–1100 °C [41]. In addition, the hexagonal ZnTiO_3_ would decompose at about 1000 °C into the metastable form Zn_2_Ti_3_O_8_ and finally to the stable cubic phase Zn_2_TiO_4_ with TiO_2−r_. These results agree with the conclusions of several authors, who indicate the presence of a low percentage of zinc oxide formation from the metastable ZnTiO_3_ which ultimately transforms (>90% conversion) to Zn_2_TiO_4_ (<900 °C) and to TiO_2−r_ from 700 °C [38,42,43,44]. The literature also indicates that obtaining either of these Ti-Zn mixed oxides as phase-pure at a low processing temperature is a challenge in materials chemistry [26,38]. On the other hand, SEM images were used to determine the shape, particle size, and morphology of the samples. The ZnTiO_3_/TiO_2_ heterostructure synthesized with a TiO_2_:ZnO molar ratio of 3:1 and calcined at 500 °C was almost spherical with an average particle size of 100 nm and, highly agglomerated. These results agree with those reported by Mehrabi et al. [42].

According to Equation (2), the calculated band gaps of ZnTiO_3_/TiO_2_ and TiO_2_ for the present study were 3.07 and 3.12 eV, respectively. These values are smaller than those reported by Li et al. [45], who synthesized *a-*TiO_2_/ZnTiO_3_ and *a-*TiO_2_ nanoparticles with a particle size smaller than 100 nm and found band gap values of 3.14 and 3.23 eV for both photocatalysts, respectively. This band gap difference may be due to the difference in particle size also known as the quantum size effect [39]. To improve conversion efficiency, a good alternative is to reduce the band gap to shift the spectral range of light absorption into the visible-light region and even the near-infrared light region [46]. Therefore, the ZnTiO_3_/TiO_2_ composite synthesized in the present study could be a promising alternative for the photocatalytic degradation of colorants in aqueous systems due to its band gap.

### 4.2. Adsorption and Photocatalytic Degradation

The photocatalytic activity of the clay-supported ZnTiO_3_/TiO_2_ was determined by decomposition under UV-C light irradiation of Methylene Blue (MB) in water following the methodology described by Ke et al. [41], with some modifications. The literature reports that UV light excites photocatalysts to decompose the organic pollutant due to the generation of electron pairs and VB holes (*e*^−^/*h*^+^) produced. The *e^−^* and *h^+^* can move in the catalyst. When they move to the surface of the catalyst, *e*^−^ reacts with O_2_ in the solution to generate *OH and *O_2_ radicals. These active species then react with MB to oxidize it and transform it into small molecules, thus achieving both purposes, decolorization and degradation of the organic matter [31,46,47,48].

The results obtained in the present study clearly indicate that ZnTiO_3_/TiO_2_ possesses excellent photocatalytic performance. These results are in line with the findings of several authors [49,50,51,52,53]. Furthermore, the ZnTiO_3_/TiO_2_ composite exhibited an important increase of the photocatalytic activity as compared with pure TiO_2_. The composite probably had better photocatalytic activity because the coupling promotes an effective separation of photo-generated electron-hole pairs between TiO_2_ and ZnTiO_3_ [41].

Ke et al. [41] reported the photocatalytic activity of *a-*TiO_2_, *a-*TiO_2_/ZnTiO_3,_ and *r-*TiO_2_/ZnTiO_3_. These authors synthesized *a-*TiO_2_/ZnTiO_3_ at 800 °C and found that it had the highest photocatalytic activity, removing approximately 80% of the dye from a 5 mg/L MB solution and under UV-C light. In the present study, ZnTiO_3_/TiO_2−a_ was synthesized at 500 °C and approximately 90% of the dye was removed from an MB solution of the same concentration and under UV-C light. This improvement is probably due to the TiO_2_:ZnO molar ratio used in the present study, which may improve the separation of the electron-hole pairs [41].

On the other hand, in agreement with other authors [54,55,56,57,58,59,60,61], the clay minerals and clay-derived materials have many diverse applications including catalysis and adsorption, which are based on their unique surface properties. The evidence of this work indicates that raw clays can be valuable supports for materials used for the removal of dyes.

The high photocatalytic activity of Clay_12_ and its photocatalytic derivatives is probably caused by the presence of natural TiO_2_ in its composition. This clay shows also a deep-red color due to the presence of a high content of Fe_2_O_3_. The photocatalytic derivatives of TiO_2_ and both Clay_1_ and Clay_2_ also show high photocatalytic activity, probably due to their mineralogical composition. Both clays are rich in SiO_2_, and according to the literature, TiO_2_/SiO_2_ photocatalysts exhibit high photocatalytic activity toward pollutant molecule decomposition [62].

In the present study, Clay_6_ shows the highest MB removal capacity. According to the literature, electrostatic interaction, complexation chemical reactions, or ionic exchange between the adsorbent and the adsorbate are some of the mechanisms that may be involved in the adsorption of MB on the surface of clay minerals [2,59]. Clay_6_ has a zero-charge point (ZCP) of 3.0. The ZCP of both TiO_2_ and ZnTiO_3_/TiO_2_ supported in Clay_6_ was 7.0. The tests were carried out at pH = 8.0; therefore, the surface of Clay_6_ was negatively charged, improving the MB cation adsorption. Moreover, according to Table 1, Clay_6_ presents several oxides, such as MgO, K_2_O, CaO, TiO_2_, MnO, and others that could promote the cationic exchange capacity of the clay to improve its adsorption capacity [63,64]. Furthermore, Clay_6_ contains Metahalloysite (M), which could also contribute to the high adsorption capacity of the clay since, according to the literature, metahalloysite has a potential application in the design and preparation of heterogeneous catalysts in procedures where the temperature did not exceed 450 °C [65].

It is important to mention that prior to illumination, MB was adsorbed onto the composites surface for 30 min in the dark and the average adsorption capacity was of 73%, 66%, 63%, 55%, and 46% approximately for ${\mathrm{Clay}}_{\overset{-}{x}}$, ZnTiO_3_/TiO_2_-${\mathrm{Clay}}_{\overset{-}{x}}$, TiO_2_-${\mathrm{Clay}}_{\overset{-}{x}}$, ZnTiO_3_/TiO_2,_ and TiO_2_, respectively. It is evident from these results that adsorption played a significant role in the degradation process of MB but is not sufficient to achieve complete degradation. Batch adsorption was therefore followed by 150 min of photocatalysis to further improve the degradation process. After 150 min of illumination, the average decolorization was improved to 89%, 82%, 85%, 92%, and 89% approximately for ${\mathrm{Clay}}_{\overset{-}{x}}$, ZnTiO_3_/TiO_2_-${\mathrm{Clay}}_{\overset{-}{x}}$, TiO_2_-${\mathrm{Clay}}_{\overset{-}{x}}$, ZnTiO_3_/TiO_2,_ and TiO_2_, respectively, which confirmed that adsorption combined with photocatalysis is an efficient process for dye degradation. These results agree with those obtained by other authors for the degradation of MB by adsorption and photocatalysis [66,67,68,69,70,71].

It is known that the separation, recovery, and reuse of the photocatalyst particles are important issues determining the future application of photocatalytic technology [72]. Therefore, in the present work, extrudates of photocatalyst-clay were prepared to facilitate the recovery of the synthesized composites. The extrudates preserved their structure during the process and no fine particles from attrition were observed; however, filters were used prior to spectrophotometer determination to avoid any interference. The use of clay extrudates with immobilized nanostructured semiconductors was an effective alternative to obtain porous photocatalysts with better active surface and adsorption capacity than isolated semiconductors, preserving their electronic and structural properties for their application in the degradation of MB under UV-C irradiation.

### 4.3. Reuse of the Supported Photocatalysts

Mechanical stability is an especially important property, which is directly related to the useful life of the supported photocatalyst. When the mechanical stability is poor, the photocatalyst will gradually flake away from the support into the reaction solution during the process; consequently, the supported photocatalyst loses its activity prematurely, and causes both secondary contamination and waste of the photocatalyst. Some investigations results showed that mechanical stability of the material is correlated with the calcination temperature [73]. Consequently, increasing the calcination temperature produces better mechanical stability, although there is an optimal calcination temperature to achieve maximum mechanical stability. In the present work, a maximum calcination temperature of the extrudates of 500 °C was used to avoid the change of crystalline phase of the synthesized photocatalysts. On average, the loss of activity of the materials did not exceed 10% at the end of the fifth cycle; thus, 500 °C is the optimum calcination temperature to achieve adequate photocatalytic activity and reuse property under the operating conditions used in this study.

Finally, the synthesized clay-supported composite reported in this paper could be an efficient alternative to remove dyes in aqueous effluents and the most probable reason is the combined effects of several factors, such as the specific surface area, the crystal size and crystallization phases, absorption capacity, photocatalytic activity, and mechanical stability. Table 2 shows the comparison of the MB degradation capacity of the main synthesized composites with other composites reported in the literature.

## 5. Conclusions

In summary, according to the results obtained, it can be concluded that the sol-gel method is useful to synthesize other perovskite-type oxides based on titanium with various architectures and novel properties. Different operating conditions were evaluated to determine the best TiO_2_/ZnO molar ratio and the optimum synthesis temperature to obtain ZnTiO_3_/TiO_2_. The results indicate that the best synthesis conditions were the following: a molar ratio of 3:1 and a calcination temperature of 500 °C. The photocatalytic materials were tested for the removal of methylene blue and, as a result, it was obtained that ZnTiO_3_/TiO_2_ had better activity than TiO_2−a_. Twelve Ecuadorian clays were characterized, some of them with exploitation potential and utility in the preparation of new materials. The photocatalysts were impregnated into the clays and their photocatalytic activity and adsorption capacity were evaluated. Clay_12_ impregnated with TiO_2−a_ showed the best photocatalytic activity. The X-ray fluorescence results showed that Clay_12_ has TiO_2_ and Fe_2_O_3_ in its composition, which could improve its activity. Clay_6_ had the best methylene blue adsorption capacity. The results of the X-ray diffraction allowed concluding that the presence of metahalloysite phase improves the adsorption capacity. In addition, Clay_6_ has various oxides with exchange cations that could improve its adsorption capacity. Thus, both clays can be used as inexpensive materials for the removal of cationic dyes from wastewater. Nevertheless, the adsorption mechanisms and adsorption reversibility should be further investigated to confirm the efficiency of these clays, as well as to elucidate which are the adequate active sites to prevent the release of cationic dyes depending on the physical-chemical conditions.

## Figures and Tables

**Table 1:** Composition (wt.%) of Ecuadorian clays.

CLAY	Al_2_O_3_	SiO_2_	MgO	P_2_O_5_	K_2_O	CaO	TiO_2_	MnO	Fe_2_O_3_	Co_3_O_4_	SnO_2_	CeO_2_
CLAY 1	13.50	66.70	2.07	0.00	1.33	1.50	0.10	0.06	0.53	0.14	0.53	0.00
CLAY 2	12.10	61.00	0.00	0.26	1.19	0.53	0.29	0.06	1.63	0.42	0.16	0.04
CLAY 3	23.10	50.40	3.17	0.23	2.51	0.22	0.41	0.08	2.45	0.61	0.00	0.12
CLAY 4	18.50	52.90	0.69	0.00	1.49	0.33	0.45	0.08	2.14	0.54	0.03	0.11
CLAY 5	21.40	40.70	2.95	0.27	0.00	0.21	0.48	0.15	4.04	1.00	0.15	0.13
CLAY 6	21.20	45.60	2.05	0.29	1.63	1.22	0.39	0.06	1.94	0.48	0.00	0.10
CLAY 7	22.30	31.90	2.65	0.39	0.09	0.84	0.72	0.12	3.63	0.89	0.44	0.12
CLAY 8	19.90	30.20	0.00	0.30	0.03	0.28	1.01	0.35	7.81	0.00	0.05	0.18
CLAY 9	13.80	40.00	0.00	0.00	0.19	0.51	0.57	0.09	3.17	0.80	0.27	0.24
CLAY 10	22.50	35.47	0.00	0.05	0.18	0.87	1.81	0.38	17.27	0.00	0.07	0.00
CLAY 11	22.20	37.80	0.00	0.19	0.00	0.08	0.30	0.08	2.48	0.61	0.04	0.04
CLAY 12	27.10	39.40	0.00	0.26	0.78	0.12	2.16	0.10	22.40	0.07	0.17	0.08

**Table 2:** MB degradation capacity of synthesized composites and other composites reported in the literature.

Material	MB Degradation Capacity (mg/g)	References
Clay_6_	19.33	This study
ZnTiO_3_/TiO_2_-Clay_6_	19.98	This study
TiO_2_-Clay_6_	18.52	This study
Clay_12_	17.11	This study
ZnTiO_3_/TiO_2_-Clay_12_	14.47	This study
TiO_2_-Clay_12_	16.85	This study
ZnTiO_3_/TiO_2_	18.40	This study
TiO_2_	17.80	This study
*a*-TiO_2_/ZnTiO_3_	16.00	[41]
*a*-TiO_2_	15.00	[41]
Red-clay	18.83	[64]
Zeolite	16.37	[74]
Natural clay	15.40	[75]
Raw Coal fly ash	5.06	[76]
Activated carbon	6.43	[77]
AC-ZnO	32.22	[78]

## References

[1] Anandan S., Ponnusamy V.K., AshokKumar M. (2020). A review on hybrid techniques for the degradation of organic pollutants in aqueous environment. Ultrason. Sonochem..

[2] Shwan D.M.S., Aziz B.K., Kaufhold S. (2019). High adsorption efficiency of topkhana natural clay for methylene blue from medical laboratory wastewater: A linear and nonlinear regression. Silicon.

[3] Omer O.S., Hussein M.A., Hussein B.H., Mgaidi A. (2018). Adsorption thermodynamics of cationic dyes (methylene blue and crystal violet) to a natural clay mineral from aqueous solution between 293.15 and 323.15 K. Arab. J. Chem..

[4] Kang S., Qin L., Zhao Y., Wang W., Zhang T., Yang L., Rao F., Song S., Lei Q. (2019). Enhanced removal of methyl orange on exfoliated montmorillonite/chitosan gel in presence of methylene blue. Chemosphere.

[5] Lu F., Astruc D. (2020). Nanocatalysts and other nanomaterials for water remediation from organic pollutants. Coord. Chem. Rev..

[6] Ang W.L., Mohammad A.W. (2020). State of the art and sustainability of natural coagulants in water and wastewater treatment. J. Clean. Prod..

[7] Jun B.-M., Al-Hamadani Y.A., Son A., Park C.M., Jang M., Jang A., Kim N.C., Yoon Y. (2020). Applications of metal-organic framework based membranes in water purification: A review. Sep. Purif. Technol..

[8] Chen M., Jafvert C.T., Wu Y., Cao X., Hankins N.P. (2020). Inorganic anion removal using micellar enhanced ultrafiltration (MEUF), modeling anion distribution and suggested improvements of MEUF: A review. Chem. Eng. J..

[9] Pan L., Ai M., Huang C., Yin L., Liu X., Zhang R., Wang S., Jiang Z., Zhang X., Zou J.-J. (2020). Manipulating spin polarization of titanium dioxide for efficient photocatalysis. Nat. Commun..

[10] Long Z., Li Q., Wei T., Zhang G., Ren Z. (2020). Historical development and prospects of photocatalysts for pollutant removal in water. J. Hazard. Mater..

[11] Nguyen D.T., Tran M.D., Van Hoang T., Trinh D.T., Pham D.T., Nguyen D.L. (2020). Experimental and numerical study on photocatalytic activity of the ZnO nanorods/CuO composite film. Sci. Rep..

[12] Karthikeyan C., Arunachalam P., Ramachandran K., Al-Mayouf A.M., Karuppuchamy S. (2020). Recent advances in semiconductor metal oxides with enhanced methods for solar photocatalytic applications. J. Alloy. Compd..

[13] Sánchez-Tovar R., Blasco-Tamarit E., Fernández-Domene R., Villanueva-Pascual M., García-Antón J.M. (2020). Electrochemical formation of novel TiO_2_-ZnO hybrid nanostructures for photoelectrochemical water splitting applications. Surf. Coat. Technol..

[14] Al-Mamun M., Kader S., Islam S., Khan M. (2019). Photocatalytic activity improvement and application of UV-TiO_2_ photocatalysis in textile wastewater treatment: A review. J. Environ. Chem. Eng..

[15] Sharma S., Kumar K., Thakur N., Chauhan S. (2019). The effect of shape and size of ZnO nanoparticles on their antimicrobial and photocatalytic activities: A green approach. Bull. Mater. Sci..

[16] Ramgir N., Bhusari R., Rawat N.S., Patil S.J., Debnath A.K., Gadkari S.C., Muthe K.P. (2020). TiO_2_/ZnO heterostructure nanowire based NO_2_ sensor. Mater. Sci. Semicond. Process..

[17] Gnanaseelan N., Latha M., Mantilla A., Sathish-Kumar K., Caballero-Briones F. (2020). The role of redox states and junctions in photocatalytic hydrogen generation of MoS_2_-TiO_2_-rGO and CeO_2_-Ce_2_Ti_3_O_8.7_-TiO_2_-rGO composites. Mater. Sci. Semicond. Process..

[18] Zalani N.M., Kaleji B.K., Mazinani B. (2019). Synthesis and characterisation of the mesoporous ZnO-TiO_2_ nanocomposite; Taguchi optimisation and photocatalytic methylene blue degradation under visible light. Mater. Technol..

[19] Jose M., Elakiya M., Dhas S.A.M.B. (2017). Structural and optical properties of nanosized ZnO/ZnTiO_3_ composite materials synthesized by a facile hydrothermal technique. J. Mater. Sci. Mater. Electron..

[20] Chen F., Yu C., Wei L., Fan Q., Ma F., Zeng J., Yi J., Yang K., Ji H. (2020). Fabrication and characterization of ZnTiO_3_/Zn_2_Ti_3_O_8_/ZnO ternary photocatalyst for synergetic removal of aqueous organic pollutants and Cr(VI) ions. Sci. Total Environ..

[21] Al-Hajji L. (2019). A comparative study on the zinc metatitanate microstructure by ball milling and solvothermal approaches. J. Struct. Chem..

[22] Baamran K.S., Tahir M. (2019). Ni-embedded TiO_2_-ZnTiO_3_ reducible perovskite composite with synergistic effect of metal/support towards enhanced H_2_ production via phenol steam reforming. Energy Convers. Manag..

[23] Chuaicham C., Karthikeyan S., Song J.T., Ishihara T., Ohtani B., Sasaki K. (2020). Importance of ZnTiO_3_ phase in ZnTi-mixed metal oxide photocatalysts derived from layered double hydroxide. ACS Appl. Mater. Interfaces.

[24] Surynek M., Spanhel L., Lapčík Ľ., Mrazek J. (2019). Tuning the photocatalytic properties of sol-gel-derived single, coupled, and alloyed ZnO-TiO_2_ nanoparticles. Res. Chem. Intermed..

[25] Müllerová J., Šutta P., Medlín R., Netrvalova M., Novak P. (2017). Optical properties of zinc titanate perovskite prepared by reactive RF sputtering. J. Electr. Eng..

[26] Surendar T., Kumar S., Shanker V. (2014). Influence of La-doping on phase transformation and photocatalytic properties of ZnTiO_3_ nanoparticles synthesized via modified sol-gel method. Phys. Chem. Chem. Phys..

[27] Acosta-Silva Y., Castanedo-Perez R., Torres-Delgado G., Méndez-López A., Zelaya-Ángel O. (2016). Analysis of the photocatalytic activity of CdS+ZnTiO_3_ nanocomposite films prepared by sputtering process. Superlattices Microstruct..

[28] Bhagwat U.O., Wu J.J., Asiri A.M., Anandan S. (2019). Synthesis of ZnTiO_3_@TiO_2_ heterostructure nanomaterial as a visible light photocatalyst. Chem. Sel..

[29] Sarkar M., Sarkar S., Biswas A., De S., Kumar P.R., Mothi E., Kathiravan A. (2020). Zinc titanate nanomaterials—Photocatalytic studies and sensitization of hydantoin derivatized porphyrin dye. NanoStruct. NanoObjects.

[30] Hadjltaief H.B., Ben Ameur S., Da Costa P., Ben Zina M., Galvez M.E. (2018). Photocatalytic decolorization of cationic and anionic dyes over ZnO nanoparticle immobilized on natural Tunisian clay. Appl. Clay Sci..

[31] Tobajas M., Belver C., Rodriguez J.J. (2016). Degradation of emerging pollutants in water under solar irradiation using novel TiO_2_-ZnO / clay nanoarchitectures. Chem. Eng. J..

[32] Belver C., Bedia J., Rodriguez J.J. (2015). Environmental Titania—Clay heterostructures with solar photocatalytic applications. Appl. Catal. B Environ..

[33] Wadhwa S., Mathur A., Pendurthi R., Singhal U., Khanuja M., Roy S.S. (2020). Titania-based porous nanocomposites for potential environmental applications. Bull. Mater. Sci..

[34] Jing G., Sun Z., Ye P., Wei S., Liang Y. (2017). Clays for heterogeneous photocatalytic decolorization of wastewaters contaminated with synthetic dyes: A review. Water Pr. Technol..

[35] Akkari M., Aranda P., Ben Rhaiem H., Amara A.B.H., Ruiz-Hitzky E. (2016). ZnO/clay nanoarchitectures: Synthesis, characterization and evaluation as photocatalysts. Appl. Clay Sci..

[36] Wu A., Wang D., Wei C., Zhang X., Liu Z., Feng P., Ou X., Qiang Y., Garcia H., Niu J.N. (2019). A comparative photocatalytic study of TiO_2_ loaded on three natural clays with different morphologies. Appl. Clay Sci..

[37] Krupskaya V.V., Zakusin S., Tyupina E.A., Dorzhieva O., Zhukhlistov A.P., Belousov P., Timofeeva M.N. (2017). Experimental study of montmorillonite structure and transformation of its properties under treatment with inorganic acid solutions. Minerals.

[38] Nolan N.T., Seery M.K., Pillai S.C. (2011). Crystallization and phase-transition characteristics of sol-gel-synthesized zinc titanates. Chem. Mater..

[39] Salavati-Niasari M., Soofivand F., Sobhani-Nasab A., Shakouri-Arani M., Faal A.Y., Bagheri S. (2016). Synthesis, characterization, and morphological control of ZnTiO_3_ nanoparticles through sol-gel processes and its photocatalyst application. Adv. Powder Technol..

[40] Alkaykh S., Mbarek A., Ali-Shattle E.E. (2020). Photocatalytic degradation of methylene blue dye in aqueous solution by MnTiO_3_ nanoparticles under sunlight irradiation. Heliyon.

[41] Ke S., Cheng X., Wang Q., Wang Y., Pan Z. (2014). Preparation of a photocatalytic TiO_2_/ZnTiO_3_ coating on glazed ceramic tiles. Ceram. Int..

[42] Mehrabi M., Javanbakht V. (2018). Photocatalytic degradation of cationic and anionic dyes by a novel nanophotocatalyst of TiO_2_/ZnTiO_3_/αFe_2_O_3_ by ultraviolet light irradiation. J. Mater. Sci. Mater. Electron..

[43] García-Ramírez E., Mondragón M., Zelaya-Ángel O. (2012). Band gap coupling in photocatalytic activity in ZnO-TiO_2_ thin films. Appl. Phys. A.

[44] Lei S., Fan H., Ren X., Fang J., Ma L., Liu Z. (2017). Novel sintering and band gap engineering of ZnTiO_3_ ceramics with excellent microwave dielectric properties. J. Mater. Chem. C.

[45] Li X., Xiong J., Huang J., Feng Z., Luo J. (2019). Novel g-C_3_N_4_/h′ZnTiO_3_-a′TiO_2_ direct Z-scheme heterojunction with significantly enhanced visible-light photocatalytic activity. J. Alloys Compd..

[46] Wang C.-L., Hwang W.-S., Chang K.-M., Kuo Y.-H., Hsi C.-S., Huang H.-H., Wang M.-C. (2011). Formation and morphology of Zn_2_Ti_3_O_8_ powders using hydrothermal process without dispersant agent or mineralizer. Int. J. Mol. Sci..

[47] Thein M.T., Pung S.-Y., Aziz A., Itoh M. (2014). The role of ammonia hydroxide in the formation of ZnO hexagonal nanodisks using sol–gel technique and their photocatalytic study. J. Exp. Nanosci..

[48] Budigi L., Nasina M.R., Shaik K., Amaravadi S. (2015). Structural and optical properties of zinc titanates synthesized by precipitation method. J. Chem. Sci..

[49] Li J., Wu N. (2015). Catalysis Science & Technology. Catal. Sci. Technol..

[50] Meshram S., Limaye R., Ghodke S., Nigam S., Sonawane S., Chikate R. (2011). Continuous flow photocatalytic reactor using ZnO—Bentonite nanocomposite for degradation of phenol. Chem. Eng. J..

[51] Kubacka A., Fernández-García M., Colón G. (2011). Advanced nanoarchitectures for solar photocatalytic applications. Chem. Rev..

[52] Pan G., Xu M., Zhou K., Meng Y., Chen H., Guo Y., Wu T. (2019). Photocatalytic degradation of methylene blue over layered double hydroxides using various divalent metal ions. Clays Clay Miner..

[53] Gayathri S., Jayabal P., Kottaisamy M., Ramakrishnan V. (2015). Synthesis of the graphene-ZnTiO_3_ nanocomposite for solar light assisted photodegradation of methylene blue. J. Phys. D Appl. Phys..

[54] Ozturk B., Pozan G.S. (2016). Promoting role of transition metal oxide on ZnTiO_3_-TiO_2_ nanocomposites for the photocatalytic activity under solar light irradiation. Ceram. Int..

[55] Wu L., Wu P., Zhu Y., Zhu N., Dang Z. (2016). Preparation and characterization of ZnTiO_3_-TiO_2_/pillared montmorillonite composite catalyst for enhanced photocatalytic activity. Res. Chem. Intermed..

[56] Wang A.-M., Bai N., Wang J.-X., Fan X.-Y., Kang Y.-H., Ma X.-R. (2018). Preparation and photocatalytic property of ZnTiO_3_/TiO_2_ heterogeneous composite material. Rengong Jingti Xuebao/J. Synth. Cryst..

[57] Zang W.-H., Ji Q.-H., Lan H.-C., Li J. (2019). Preparation of ZnTiO_3_/TiO_2_ photocatalyst and its mechanism on photocatalytic degradation of organic pollutants. Huanjing Kexue/Environ. Sci..

[58] El Mouzdahir Y., Elmchaouri A., Mahboub R., Gil A., Korili S.A. (2007). Adsorption of methylene blue from aqueous solutions on a Moroccan clay. J. Chem. Eng. Data.

[59] Almeida C., Debacher N.A., Downs A., Cottet L., Mello C. (2009). Removal of methylene blue from colored effluents by adsorption on montmorillonite clay. J. Colloid Interface Sci..

[60] Aguiar J.E., Cecilia J.A., Tavares P.A.S., Azevedo D.C.S., Rodríguez Castellón E., Lucena S.M.P., Silva Junior I.J. (2017). Applied clay science adsorption study of reactive dyes onto porous clay heterostructures. Appl. Clay Sci..

[61] Ruiz-Hitzky E., Aranda P., Akkari M., Khaorapapong N., Ogawa M. (2019). Photoactive nanoarchitectures based on clays incorporating TiO_2_ and ZnO nanoparticles. Beilstein J. Nanotechnol..

[62] Zhang Z., Hu L., Zhang H., Yu L., Liang Y. (2020). Large-sized nano-TiO_2_/SiO_2_ mesoporous nanofilmconstructed macroporous photocatalysts with excellent photocatalytic performance. Front. Mater. Sci..

[63] Susana S., Vercelone Z., Sham E.L., Mónica E., Torres F. (2015). Caracterización superficial y textural de organoarcillas pilareadas con TiO_2_ Surface and textural characterization of TiO_2_ pillared organoclays. Rev. Mater..

[64] Bentahar Y., Draoui K., Hurel C., Ajouyed O., Khairoun S., Marmier N. (2019). Physico-chemical characterization and valorization of swelling and non-swelling Moroccan clays in basic dye removal from aqueous solutions. J. Afr. Earth Sci..

[65] Carrillo A.M., Urruchurto C.M., Carriazo J.G., Moreno S., Molina R.A. (2014). Structural and textural characterization of a Colombian halloysite. Rev. Mex. Ing. Quim..

[66] Laysandra L., Sari M.W.M.K., Soetaredjo F.E., Foe K., Putro J.N., Kurniawan A., Ju Y.-H., Ismadji S. (2018). Adsorption and photocatalytic performance of bentonite-titanium dioxide composites for methylene blue and rhodamine B decoloration. Heliyon.

[67] Chen Y., Xiang Z., Wang D., Kang J., Qi H. (2020). Effective photocatalytic degradation and physical adsorption of methylene blue using cellulose/GO/TiO_2_ hydrogels. RSC Adv..

[68] Irani M., Mohammadi T., Mohebbi S. (2017). Photocatalytic degradation of methylene blue with ZnO nanoparticles; a joint experimental and theoretical study. J. Mex. Chem. Soc..

[69] Kurajica S., Minga I., Blazic R., Muzina K., Tominac P. (2018). Adsorption and degradation kinetics of methylene blue on as-prepared and calcined titanate nanotubes. Athens J. Sci..

[70] Makama A.B., Salmiaton A., Saion E.B., Choong T.S.Y., Abdullah N. (2016). Synthesis of CdS sensitized TiO_2_ photocatalysts: Methylene blue adsorption and enhanced photocatalytic activities. Int. J. Photoenergy.

[71] Shahid M., El Saliby I., McDonagh A., Chekli L., Tijing L.D., Kim J.-H., Shon H.K. (2016). Adsorption and photocatalytic degradation of methylene blue using potassium polytitanate and solar simulator. J. Nanosci. Nanotechnol..

[72] Belver C., Hinojosa M., Bedia J., Tobajas M., Alvarez M.A., Rodríguez-González V. (2017). Electronic supplementary information ag-coated heterostructures of ZnO-TiO_2_/ delaminated montmorillonite as solar photocatalysts. Materials.

[73] Shi J.W., Chen S.H., Wang S.M., Ye Z.L., Wu P., Xu B. (2010). Favorable recycling photocatalyst TiO_2_/CFA: Effects of calcination temperature on the structural property and photocatalytic activity. J. Mol. Catal. A Chem..

[74] Han R., Zhang J., Han P., Wang Y., Zhao Z., Tang M. (2009). Study of equilibrium, kinetic and thermodynamic parameters about methylene blue adsorption onto natural zeolite. Chem. Eng. J..

[75] Sahoo S., Uma, Banerjee S., Sharma Y.C. (2013). Application of natural clay as a potential adsorbent for the removal of a toxic dye from aqueous solutions. Desalin. Water Treat..

[76] Li H., Dai M., Dai S., Dong X., Li F. (2018). Methylene blue adsorption properties of mechanochemistry modified coal fly ash. Hum. Ecol. Risk Assess. Int. J..

[77] Nayeri D., Mousavi S.A., Fatahi M., Almasi A., Khodadoost F. (2019). Dataset on adsorption of methylene blue from aqueous solution onto activated carbon obtained from low cost wastes by chemical-thermal activation—Modelling using response surface methodology. Data Br..

[78] Nourmoradi H., Ghiasvand A., Noorimotlagh Z. (2014). Removal of methylene blue and acid orange 7 from aqueous solutions by activated carbon coated with zinc oxide (ZnO) nanoparticles: Equilibrium, kinetic, and thermodynamic study. Desalin. Water Treat..

